# Process method of Si_3_N_4_ ceramic brazing sealed cavity for high-temperature application

**DOI:** 10.3389/fchem.2022.1019822

**Published:** 2022-09-27

**Authors:** Chen Li, Zhihong Fang, Boshan Sun, Jijun Xiong, Aodi Xu, Ximing Guo, Yingping Hong

**Affiliations:** ^1^ Science and Technology on Electronic Test and Measurement Laboratory, North University of China, Taiyuan, China; ^2^ State Key Laboratory of Dynamic Measurement Technology, North University of China, Taiyuan, China; ^3^ Key Laboratory of Instrumentation Science and Dynamic Measurement, Ministry of Education, North University of China, Taiyuan, China

**Keywords:** Si_3_N_4_ ceramic, vacuum brazing, brazing interface, sealed cavity, high temperature

## Abstract

The process method of a Si_3_N_4_ ceramic sealed cavity is realized by vacuum brazing and chemical reaction at 1,100°C and 0.5 MPa pressure. Through the combination of Si_3_N_4_ ceramic polishing and thinning, inductively coupled plasma etching, and high-temperature metal filler (Ti-Zr-Cu-Ni) brazing process, a vacuum-sealed cavity suitable for high-temperature environments was prepared. The cross section of the bonding interface was characterized by scanning electron microscope (SEM) and energy dispersive spectrometer (EDS), which indicated that the two Si_3_N_4_ ceramic were well bonded, the cavity structure remained intact, and the bonding interface strength exceeded 5.13 MPa. Furthermore, it retained its strong bonding strength after in high-temperature environments of 1,000, 1,050, and 1,100°C for 1 h. This indicates that a brazed vacuum-sealed cavity can be used in high-temperature environments. Through the proposed method, pressure sensor that can withstand high temperatures can be developed.

## Introduction

In high-temperature environments, *in situ* and accurate pressure measurements are required for industrial applications, such as energy extraction, environmental monitoring, turbine engines, high-speed aircraft, and other aerospace applications (Lü et al., 2018; Chen et al., 2022; Li et al., 2022; Zhang et al., 2022), to improve component conditions and ensure engine reliability (Bonham et al., 2017; Miura et al., 2017; Han et al., 2020; He et al., 2021). Materials are crucial as the cornerstone of pressure sensors, as sensors made of ordinary materials cannot operate optimally in high-temperature environments. Therefore, high-temperature-resistant materials are preferred, and by using these, developing pressure sensors that can be used in ultra-high temperature environment. It is crucial and significant to develop an original test of pressure parameters in high-temperature environments (Vysotina et al., 2021).

The operating temperatures of conventional silicon pressure sensors and some silicon-on-insulator (SOI)-based pressure sensors are generally in the range of 200–300°C, which cannot be adopted in high-temperature environments (Cheng et al., 2021; Tang et al., 2021). Hence, ceramic pressure sensors have been developed (Li J. et al., 2019; Meng et al., 2021). The technology of these sensors is mature and they are small-sized. However, the operating temperatures of these sensors generally do not exceed 500°C, imposing significant limitations when operating in higher temperature environments (Naderi and Moghaddam, 2020; Salvatori et al., 2020; Abdel-Motaleb and Dittakavi, 2021). Most LC-type high temperature pressure sensors are fabricated using either a low-temperature co-fired ceramic (LTCC) or high-temperature co-fired ceramic (HTCC) technology (Lin et al., 2018; Zhu et al., 2018; Raynaud et al., 2021; Zhang et al., 2021; Seek and Makarovi, 2022). In these methods, sensors are using combined processes of multilayer ceramics and laminations, high-temperature sintering, and screen-printing processes, however, the manufacturing process is complex. In this process, the embedded sealed cavity is easily deformed or even collapses, thereby resulting production difficulties. Therefore, in recent years, several researchers have developed pressure sensors from some high-temperature resistant materials (such as sapphire/sapphire and MgO/MgO, etc.) *via* direct process bonding, which can be applied to high-temperature environments. However, its preparation process is complicated and expensive (Li et al., 2017; Li W. et al., 2019; Liu et al., 2019; Liu et al., 2020). Si_3_N_4_ ceramic has excellent properties such as high hardness and strength, low thermal expansion coefficient, optimal oxidation resistance, and high corrosion resistance (Gu, 2004; Usherenko et al., 2019; Xiao and Han, 2019; Ye et al., 2021), in particular, hot-pressed Si_3_N_4_ ceramic, which is extremely resistant to high temperatures, can be utilized as a high-temperature sensor core substrate while preparing high temperature pressure sensor cavity.

In this study, we propose a high-temperature pressure sensor sealed cavity fabrication method based on Si_3_N_4_ ceramic, fabricated by etching Si_3_N_4_ ceramic *via* metal masks and brazing Si_3_N_4_ ceramic with a high-temperature active brazing filler (Ti-Zr-Cu-Ni). For the brazing filler, joints brazed with mature and widely used Ag-Cu-Ti (Deng et al., 2021) brazing fillers exhibit excellent mechanical properties. However, owing to the limitation of the working temperature, their application in high-temperature environments is limited. It is necessary to select appropriate refractory metals to maximize excellent high-temperature performance of Si_3_N_4_ ceramic. Accordingly, Cu-Ni is a more suitable choice, than noble metals. Nevertheless, to ensure that the brazing filler can react with the ceramic, the active metal Ti-Zr was selected to achieve optimal wettability with the ceramic. Furthermore, tensile tests and microstructural observations of the bonded interface were performed on the brazed samples. The connection method proposed in this study can realize the high-strength connection of Si_3_N_4_ ceramic/Si_3_N_4_ ceramic and form a well-sealed cavity, thus providing a basis for the next production of pressure sensors. Compared with conventional SOI-based pressure sensors, this type of ceramic can be used in higher-temperature environments. Furthermore, compared with HTCC technology and directly bonded technology, this type of method has a simpler manufacturing process, is less expensive, and provides broad application prospects in high temperature pressure sensors.

## Experiment

### Polishing and thinning of samples

**FIGURE 1:**
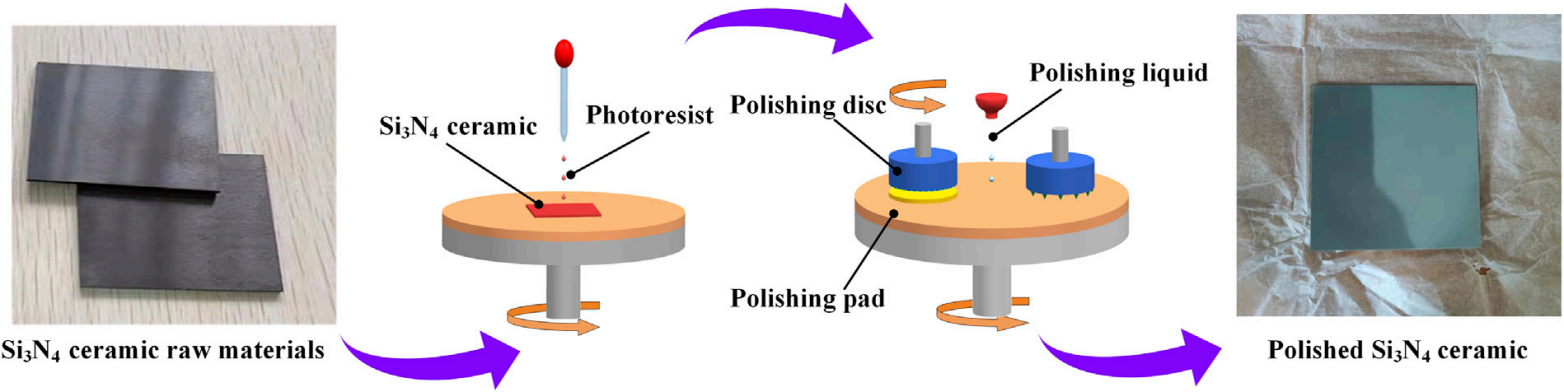
Polishing process of Si_3_N_4_ ceramic.

The raw materials used in this experiment are Si_3_N_4_ ceramic with a purity of 99.9%. In the manufacturing process of the sealed cavity, three key processes are mainly included: the Si_3_N_4_ ceramic polishing reduction process, Si_3_N_4_ ceramic etching process, and Si_3_N_4_ ceramic bonding process. According to the designed preparation process, first of all, the thickness and roughness of the sample ceramics should be reduced. Because the close contact between the sample ceramics cannot be guaranteed if the roughness is too large, and so that the brazing quality cannot be guaranteed. At the same time, it will affect the high-temperature brazing filler metal to fully expand on the ceramics, resulting in loose connection. The process of thinning can ensure the flatness of the sample ceramics. The Si_3_N_4_ ceramic material is harder. The abrasive used in the grinding process is boron carbide material. In the grinding process of chemical machinery, while the ceramic sheet is flattened, residual stress will be introduced on the surface of the chip due to mechanical grinding. The thinned Si_3_N_4_ ceramic was subjected to silicon oxide suspension polishing to satisfy the surface appearance requirements of the subsequent brazing process. The root mean square roughness was approximately 0.2 μm measured by atomic force microscopy (AFM). Its process flow is illustrated in Figure 1.

### Chemical etching process of Si_3_N_4_ ceramic

Subsequently, a metal mask was utilized for etching. The specific process is as follows:

1) Sputtering aluminum film: A 3-μm thick aluminum film was sputtered onto a Si_3_N_4_ ceramic substrate *via* physical vapor deposition (PVD), the power and Ar flow adopted were 3 kW and 20 standard cubic centimeter per minute (sccm), respectively. The sputtering rate is 6 nm/s;

2) Spin coating photoresist: The positive photoresist AZ6130 was evenly applied on the Si_3_N_4_ ceramic by rotating the gelatinizer, with an average glue speed of 500 r/min for 10 s, and 3,000 r/min for 60 s, to obtain a layer of photoresist;

3) Patterned exposure: The Si_3_N_4_ ceramic coated with photoresist were placed upward, and the mask plate with lithography pattern placed downward into the lithography machine to ensure that the mask plate and the silicon wafer were fixed. The pattern on the mask printed by the was transferred to the Si_3_N_4_ ceramic at a ratio of 1: 1;

4) Corroding aluminum: The exposed aluminum was subsequently corroed using phosphoric acid (H_3_PO_4_) at 85°C;

5) ICP etching Si_3_N_4_ ceramic: After etching starts, sulfur hexafluoride (SF_6_) and trifluoromethane (CHF_3_) were used as etching gases, oxygen (O_2_) was used to clean the chamber and took away the exhaust gas produced by etching, helium (He) was used as protective gas, and the pressure of the process chamber was set to 12 mTorr with the etching power 2500 W;

6) Etched Si_3_N_4_ ceramic: After etching, the remaining aluminum film was etched away;

7) Coating brazing filler: Apply high-temperature brazing filler on the etched the samples;

8) Prepare brazing samples: Complete the preparation of samples.

After the experimental samples were prepared, they were brazed using a filler metal coating. As ordinary components do not wet the surface of Si_3_N_4_ ceramic, it is necessary to add a small amount of active elements to the conventional metal brazing technology, which promotes the wetting of the brazing filler on the ceramic surface. In this study, Ti and Zr were adopted as active elements to achieve wetting *via* the following reactions.

Si3N4+4Zr=4ZrN+3Si (1)

Si3N4+4Ti=4TiN+3Si (2)

**FIGURE 2:**
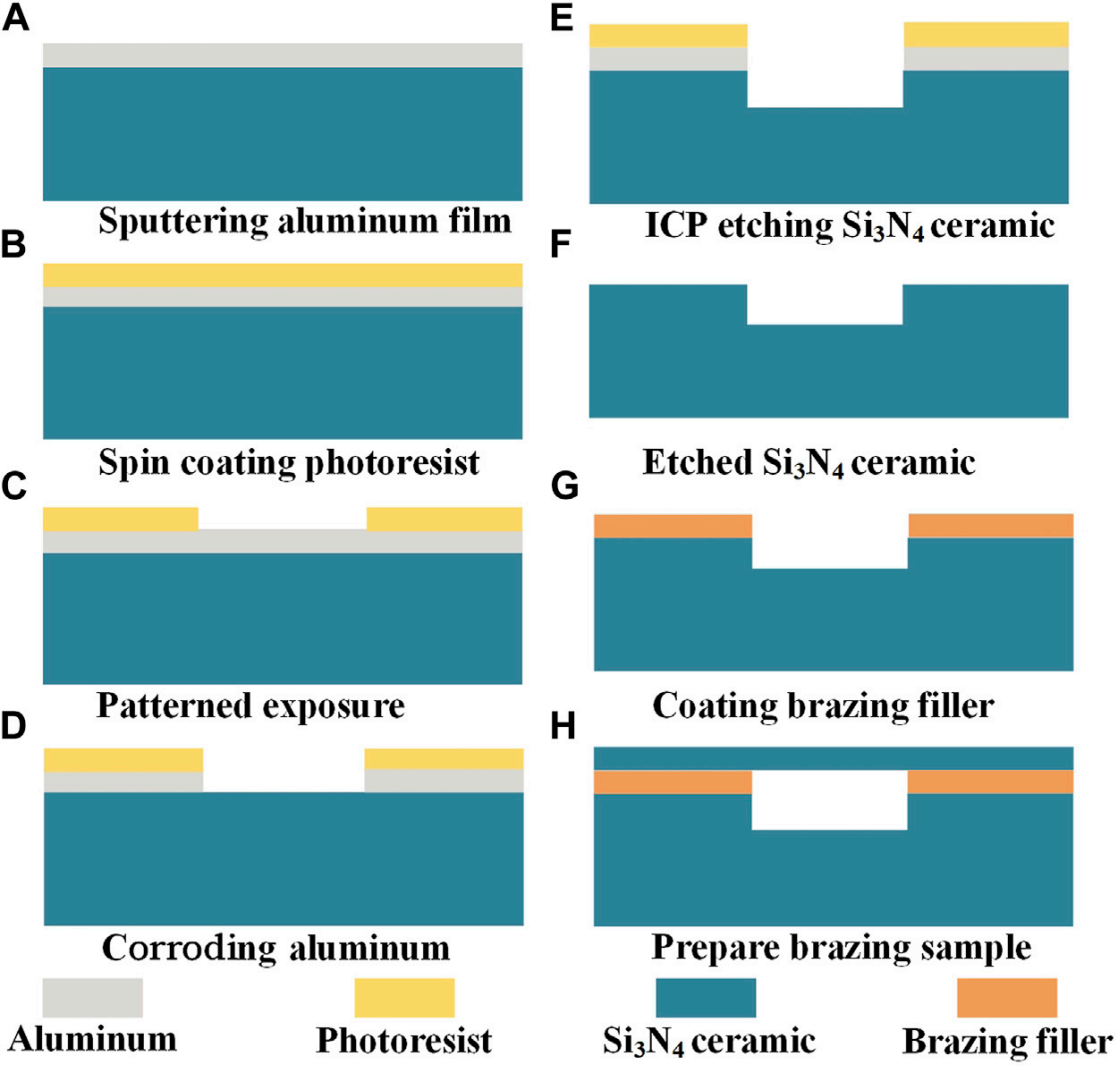
Etching process and brazing process of Si_3_N_4_ ceramic: **(A)** Sputtering aluminum film. **(B)** Spin coating photoresist. **(C)** Patterned exposure. **(D)** Corroding aluminum. **(E)** ICP etching Si_3_N_4_ ceramic. **(F)** Etched Si_3_N_4_ ceramic. **(G)** Coating brazing filler. **(H)** Prepare brazing samples.

We selected a Ti-Zr-Cu-Ni brazing filler with optimal stability and oxidation resistance to maximize the excellent high-temperature performance of Si_3_N_4_ ceramic in the active brazing of Si_3_N_4_ ceramic. Connected Si_3_N_4_ ceramic remain significantly high-temperature resistant and can resist oxidation resistance and corrosion resistance at high temperatures. First, acetone and alcohol were sequentially used in an ultrasonic cleaner for 20 min in sequence, and finally cleaned with deionized water (DI) and dried with high-purity nitrogen. The prepared brazing material was then applied to the Si_3_N_4_ ceramic, thus completing the preparation of the pre-brazing samples. Figure 2 illustrates the Si_3_N_4_ ceramic etching process and sample preparation before high-temperature active brazing.

### High-temperature active brazing process

**FIGURE 3:**
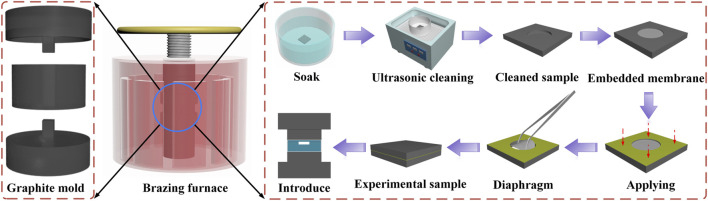
Schematic diagram of the brazing furnace and sample processing process diagram.

The prepared samples were then placed in a graphite mold. The mold comprises an upper indenter, a sleeve, and a lower indenter. Owing to the pressure provided by the upper indenter, ceramic tightness can be guaranteed during the brazing process. Contact with lower indenter was combined to apply pressure evenly on the sample surface. The assembled mold was then introduced into a brazing furnace for high-temperature brazing. The brazing furnace device rapidly heated the Si_3_N_4_ ceramic to be welded using electrodes, with the heating speed controlled by controlling the power of the workpiece with a pressure pump and a header displacement control system. Simultaneously the hot-pressing furnace table was provided with a column connection on the movable beam to ensure the verticality of the compression. A schematic diagram of the brazing furnace and the sample processing process is presented in the Figure 3. To avoid contaminating the cavity, during the coating process of the brazing filler, a membrane with the same size as the cavity was utilized to cover the cavity, and after the coating was completed, the membrane was gently removed with tweezer.

**FIGURE 4:**
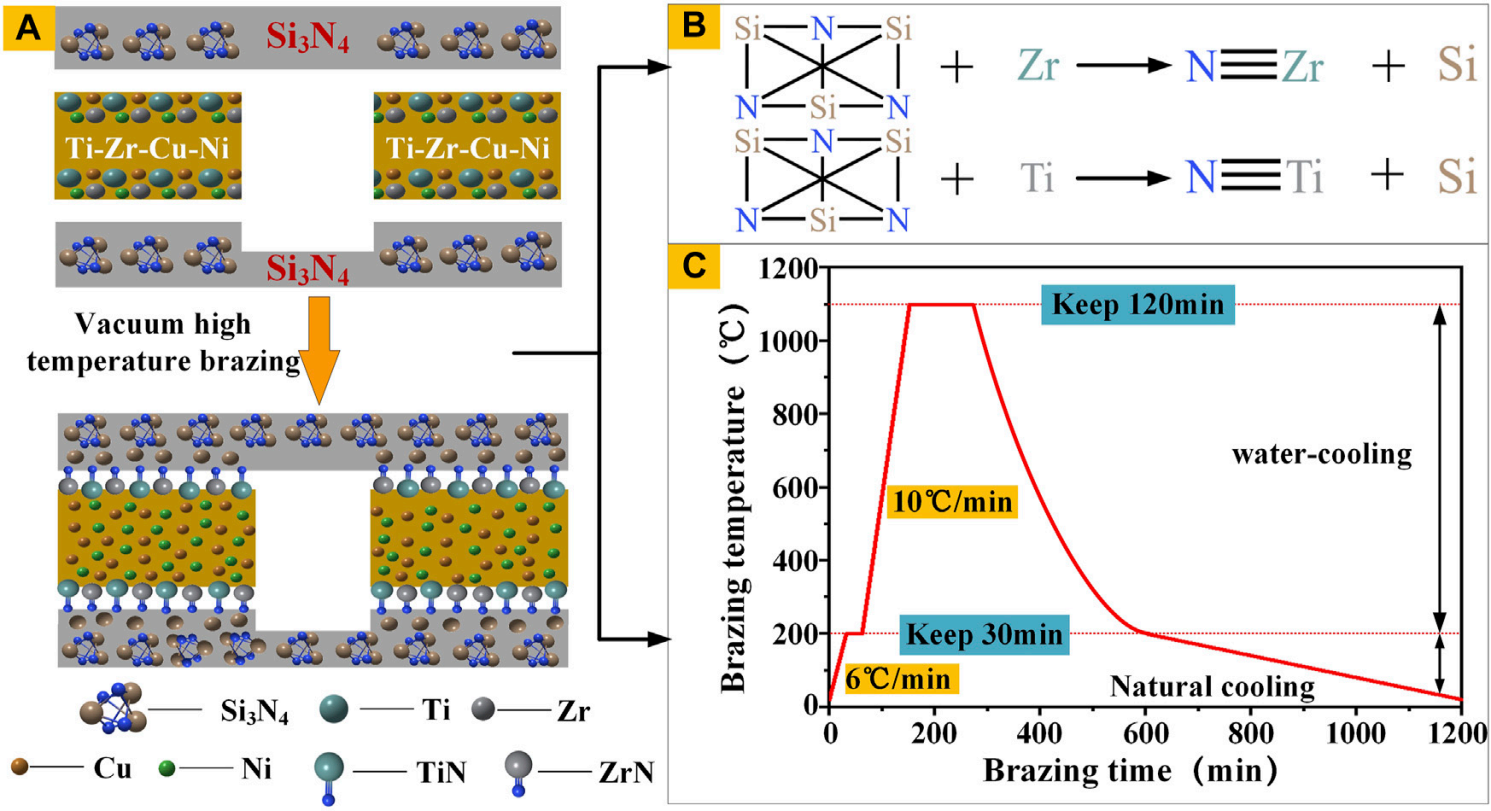
**(A)** Brazing process **(B)** High temperature brazing principle; **(C)** Heating process curve diagram.

Finally, the assembled mold was introduced into a brazing furnace for high-temperature brazing. When the vacuum degree in the vacuum heating furnace reached approximately 2.8 × 10^−3^ Pa, the sample to be welded was heated to 200°C at a rate of 6°C/min, and maintained for 30 min. The sample was then heated to the brazing temperature at a rate of 10°C/min, and maintained for a certain time. During the heat preservation process, a pressure of 0.5 MPa was applied on the sample simultaneously. Figures 4A,B illustrate the brazing process and its principle.

After brazing, the temperature was reduced to 200°C at a rate slightly lower than the heating rate, and then cooled in the furnace. Figure 4C shows the complete heating process curve. After cooling the vacuum furnace to room temperature, the Si_3_N_4_ ceramic samples were removed.

## Results and discussion

### Tensile test

**FIGURE 5:**
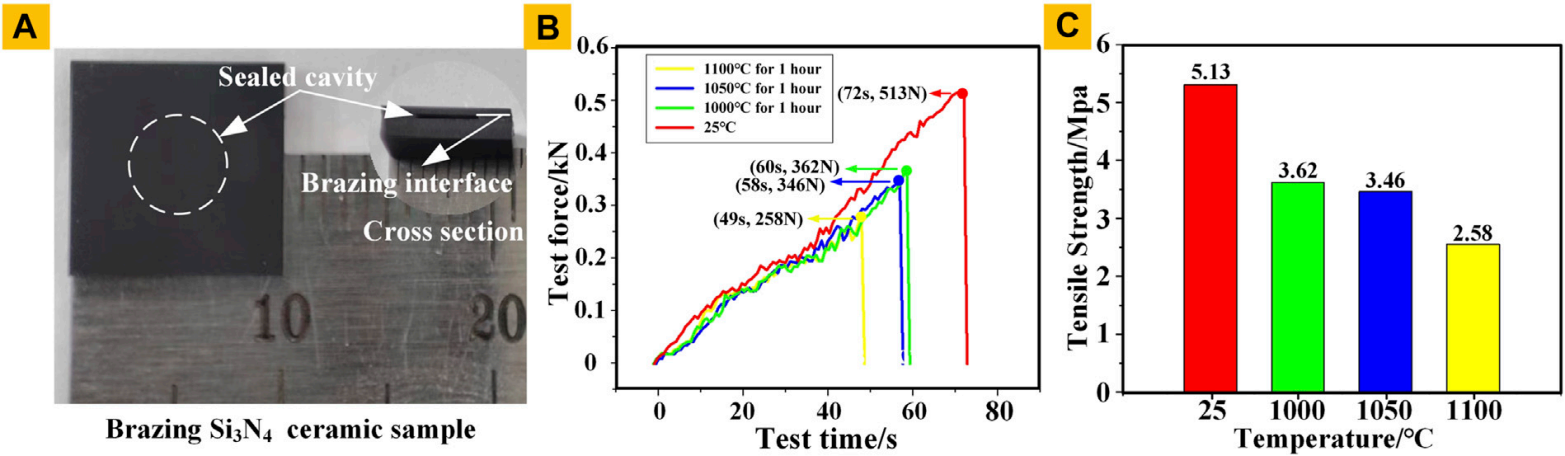
**(A)** Brazed Si_3_N_4_ ceramic sample **(B)** The curves of test time and test force after different temperature; **(C)** Tensile strength tests after different temperature.

Tensile tests were conducted on the brazed ceramic samples to evaluate the brazing strength of the Si_3_N_4_ ceramic brazing interface. Form Figure 5A, the sealed cavity of the brazed sample remains intact, and the sample is adhesively fixed to two vertical aluminum blocks and then mounted on the vertical fixture of the stretching machine. The final bonded interface fractured under a load of 513 N, and the bonded interface remained intact, thus indicating that the tensile strength of the bonded interface exceeded 5.13 MPa. To test the connection strength after the high temperature experiment, we placed the Si_3_N_4_ ceramic samples in a high temperature furnace at 1,000, 1,050, and 1,100°C for 1 h, then they were fixed on the tensile machine with adhesive for tensile test, and finally they were pulled apart under the tensile force of 362, 346, and 258 N, respectively. The curves of test time and test force are illustrated in Figure 5B. The tensile strength tests after different temperature are 5.13, 3.62, 3.46, and 2.58 MPa, as presented in Figure 5C. These values exceed the joint strength required to fabricate some pressure sensors.

### Scanning electron microscope characterization

**FIGURE 6:**
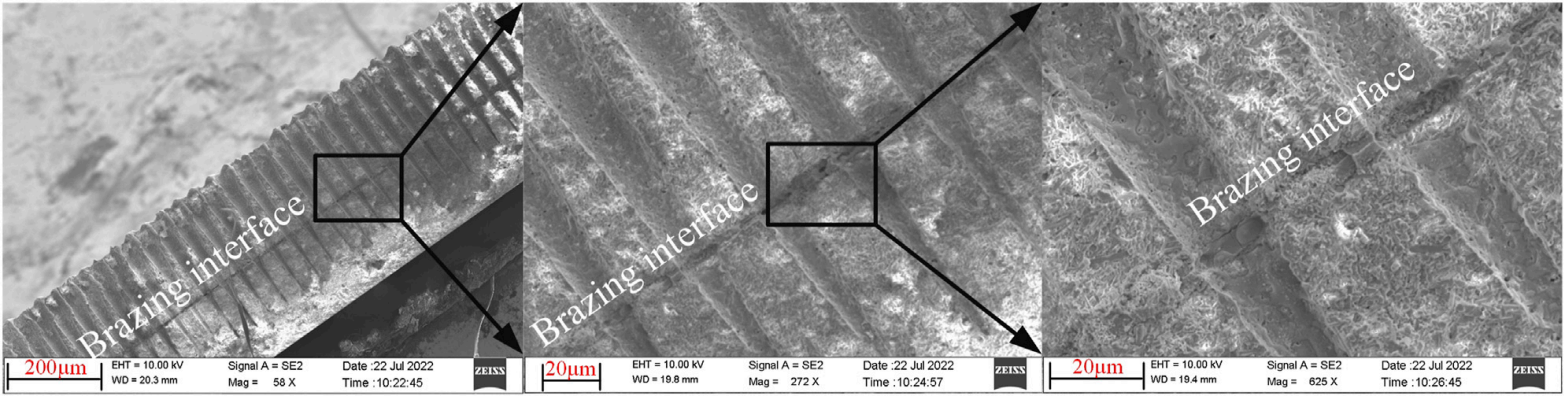
SEM images of the cross section of the brazed interface at different magnifications.

To investigate the quality of the internal connections, the samples were cut along the cross sections, which were then gold-sprayed and imaged under a scanning electron microscope (SEM). Figure 6 presents the SEM images of the brazed sample’s cross section at different magnifications. It can be observed that the brazing interface is neat, smooth and tightly connected.

### Energy dispersive spectrometer characterization

**FIGURE 7:**
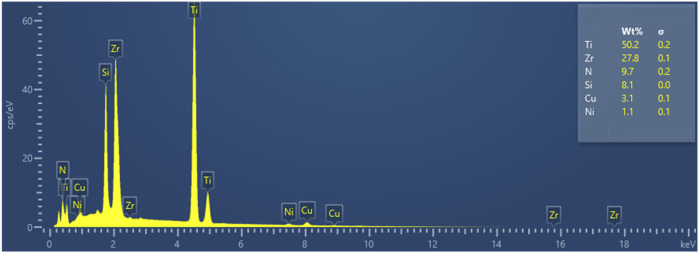
The Energy Dispersive Spectrometer (EDS) analysis results of the brazed interface.

The qualitative analysis of the bonding interface elements by the energy dispersive spectrometer (EDS) attached to the electron microscope was carried out. The result presents in Figure 7 indicates that there was no formation of new elements in the bonding interface.

## Conclusion

In this study, a process method of Si_3_N_4_ ceramic sealed cavity was proposed. The cavity was fabricated by combining polishing and thinning techniques with chemical etching. The vacuum-sealed cavity structure was completed using the chemical reaction between the materials through a high-temperature active brazing process. The tensile strength of the brazed samples exceeds 5.13 MPa, and can maintain high brazed strength after high-temperature environments of 1,000, 1,050, and 1,100°C for 1 h. In addition, the SEM and EDS characterizations of the brazing interface verify that the brazing quality is optimal, no impurities are introduced, and the cavity structure is complete. Accordingly, the brazing interface was uniform and seamless. This method obtains a tightly sealed cavity, which can be adopted for fabricating high-temperature pressure sensors.

## Data Availability

The original contributions presented in the study are included in the article/Supplementary Material, further inquiries can be directed to the corresponding author.

## References

[B1] Abdel-Motaleb I. Dittakavi S. (2021). Sic thz sensors for harsh environment applications. OJAPr. 9 (3), 45–55. 10.4236/ojapr.2021.93004

[B2] Bonham C. Thorpe S. J. Erlund M. N. Stevenson R. J. (2017). Combination probes for stagnation pressure and temperature measurements in gas turbine engines. Meas. Sci. Technol. 29 (1), 015002. 10.1088/1361-6501/aa925c

[B3] Chen S. Wang Y. Fei B. Long H. Wang T. Zhang T. (2022). Development of a flexible and highly sensitive pressure sensor based on an aramid nanofiber-reinforced bacterial cellulose nanocomposite membrane. Chem. Eng. J. 430, 131980. 10.1016/j.cej.2021.131980

[B4] Cheng L. Wang R. Hao X. Liu G. (2021). Design of flexible pressure sensor based on conical microstructure pdms-bilayer graphene. Sensors 21 (1), 289. 10.3390/s21010289 PMC7796102 33406679

[B5] Deng J. Li H. Deng M. Xia X. Liu J. Fan S. (2021). Brazing of graphite and stainless steel with Ag-Cu-Ti filler: Effects of brazing process parameters on microstructure and mechanical properties. Mat. Today Commun. 28, 102544. 10.1016/j.mtcomm.2021.102544

[B6] Gu D. (2004). Evolution of intergranular boundaries and phases in sic and Si_3_N_4_ ceramics under high temperature deformation: Case studies by analytical tem. Int. J. Mater. Res. 95 (4), 271–274. 10.3139/146.017949

[B7] Han R. Bohn C. Bauer G. (2020). Virtual engine in-cylinder pressure sensor for automobiles and agricultural tractors. IFAC-PapersOnLine 53 (1), 543–548. 10.1016/j.ifacol.2020.06.091

[B8] He X. Ran Z. Ding Z. Shao T. Gan L. Yu M. (2021). “Fast-response high-temperature all-fiber fabry-perot dynamic pressure sensor for internal combustion engine,” in Asia Communications and Photonics Conference 2021 (Shanghai, China: IEEE). C.paper W4A.5. 10.1364/ACPC.2021.W4A.5

[B9] Li J. Jia P. Fang G. Wang J. Qian J. Ren Q. (2022). Batch-producible all-silica fiber-optic fabry–perot pressure sensor for high-temperature applications up to 800°c. Sensors Actuators A Phys. 334, 113363. 10.1016/j.sna.2022.113363

[B10] Li J. Jiang Y. Li H. Liang X. Zhang D. Liu D. (2019). Direct bonding of silicon carbide with hydrofluoric acid treatment for high-temperature pressure sensors. Ceram. Int. 46 (3), 3944–3948. 10.1016/j.ceramint.2019.10.123

[B11] Li W. Liang T. Chen Y. Jia P. Xiong J. Hong Y. (2017). Interface characteristics of sapphire direct bonding for high-temperature applications. Sensors 17 (9), 2080. 10.3390/s17092080 PMC5621027 28892010

[B12] Li W. Liang T. Jia P. Lei C. Xiong j. Li Y. (2019). Fiber-optic fabry–perot pressure sensor based on sapphire direct bonding for high-temperature applications. Appl. Opt. 58 (7), 1662. 10.1364/AO.58.001662 30874197

[B13] Lin L. Ma M. Zhang F. Liu F. Liu Z. Li Y. (2018). Integrated passive wireless pressure and temperature dual-parameter sensor based on LTCC technology. Ceram. Int. 44, S129–S132. 10.1016/j.ceramint.2018.08.159

[B14] Liu J. Jia P. Chen X. Liang T. Xiong J. Liu W. (2019). Surface characterization of patterning on mgo single crystals using wet chemical etching process to advance mems devices. J. Micromech. Microeng. 30 (1), 015001. 10.1088/1361-6439/ab504d

[B15] Liu J. Jia P. Li J. Feng F. Xiong J. (2020). Hydrophilic direct bonding of mgo/mgo for high-temperature mems devices. IEEE Access 8 (99), 1. 10.1109/ACCESS.2020.2985750

[B16] Lü X. Jiang J. Wang H. Gao Q. Zhao S. Li N. (2018). Sensitivity-compensated micro-pressure flexible sensor for aerospace vehicle. Sensors 19 (1), 72. 10.3390/s19010072 PMC6338916 30585229

[B17] Meng Q. Lu Y. Wang J. Chen D. Chen J. (2021). A piezoresistive pressure sensor with optimized positions and thickness of piezoresistors. Micromachines 12 (9), 1095. 10.3390/mi12091095 34577738 PMC8469529

[B18] Miura K. Owashi M. Mihara Y. (2017). “High durability thin-film pressure sensor development for engine sliding surface,” in The Proceedings of the International symposium on diagnostics and modeling of combustion in internal combustion engines, Okayama, Japan (The Japan Society of Mechanical Engineers), C314. 10.1299/jmsesdm.2017.9.C314

[B19] Naderi N. Moghaddam M. (2020). Ultra-sensitive UV sensors based on porous silicon carbide thin films on silicon substrate. Ceram. Int. 46 (9), 13821–13826. 10.1016/j.ceramint.2020.02.173

[B20] Raynaud J. Pateloup V. Bernard M. Gourdonnaud D. Passerieux D. Cros D. (2021). Hybridization of additive manufacturing processes to build ceramic/metal parts: Example of HTCC. J. Eur. Ceram. Soc. 41 (3), 2023–2033. 10.1016/j.jeurceramsoc.2020.10.032

[B21] Salvatori S. Ponticelli G. S. Pettinato S. Genna S. Guarino S. (2020). High-pressure sensors based on laser-manufactured sintered silicon carbide. Appl. Sci. (Basel). 10 (20), 7095. 10.3390/app10207095

[B22] Seek A. Makarovi K. (2022). Metallization, material selection, and bonding of interconnections for novel ltcc and htcc power modules. Materials 15 (3), 1036. 10.3390/ma15031036 35160980 PMC8839124

[B23] Tang X. Tian J. Zhao J. Jin Z. Liu Y. Liu J. (2021). Structure design and optimization of SOI high-temperature pressure sensor chip. Microelectron. J. 118, 105245. 10.1016/j.mejo.2021.105245

[B24] Usherenko S. Ovtchinikov V. Shmuradko V. Kirshina N. (2019). Ceramics based on reactional connected silicon nitride used for foundry crucibles. Litʹë i Metall. 0 (2), 26–30. 10.21122/1683-6065-2000-2-26-30

[B25] Vysotina E. Rizakhanov R. Sigalaev S. Polushin N. Mishanin A. (2021). Sensitive elements of high temperature pressure sensors formed from a doped polycrystalline diamond. Mater. Sci. Forum 1031, 178–183. 10.4028/www.scientific.net/msf.1031.178

[B26] Xiao C. Han B. (2019). Study on the effect of bonding process of silicon nitride-based ceramics with Yb-β/Glass composite solders on the structure and properties of joints. J. Inst. Eng. India. Ser. D. 100 (2), 291–299. 10.1007/s40033-019-00190-5

[B27] Ye C. Liu Y. Wang C. Wei W. Jia H. Liu B. (2021). Investigation on thermal conductivity and mechanical properties of Si_3_N_4_ ceramics via one-step sintering. Ceram. Int. 47 (23), 33353–33362. 10.1016/j.ceramint.2021.08.238

[B28] Zhang X. Chen N. Wu J. Wei J. He N. Li L. (2021). Rapid fabrication of surface microstructures on aln htcc substrate by chemically assisted laser ablation. Ceram. Int. 47 (19), 27598–27608. 10.1016/j.ceramint.2021.06.184

[B29] Zhang Y. Jiang Y. Cui Y. Feng X. Hu J. (2022). An all-fiber diaphragm-based extrinsic fabry–perot sensor for the measurement of pressure at ultra-low temperature. Meas. Sci. Technol. 33 (5), 055117. 10.1088/1361-6501/ac51f0

[B30] Zhu Y. Y. Yang Y. J. Chen J. X. (2018). High-performance bandpass filter using htcc stepped-impedance resonators. IET Microw. Antennas Propag. 12 (1), 56–62. 10.1049/iet-map.2017.0422

